# Selections and Abstracts

**Published:** 1915-08

**Authors:** 


### SELECTIONS AND ABSTRACTS

(From the Medical Council, Sept. 15., Philadelphia, Pa.)

### SUSPECTED DIPHTHERIA: ITS PROPHYLACTIC TREATMENT.

By M. Clayton Thrush, Ph.M., M.D., 3705 Spring Garden Street, Philadelphia, Pa.

Diphtheria is such an important disease, and so much original investigation has been bestowed upon it during the past decade, that any attempt to add anything of decided value would appear almost superfluous. Hence my main object in presenting the present paper is to emphasize the great value of a certain remedy in the prophylactic treatment of suspected cases. The subject is so extensive, and so much might be written, that only one phase of the prophylactic treatment will be considered in detail. Every general practitioner treats many cases of "sore throat," and those of us who practice in the larger cities find that a large percentage of these cases, especially in children, are diptheriatic in character.

The treatment that I shall now describe is the one that I have used in my last two hundred cases of suspected diphtheria, and with the most gratifying results. In discussing the symptoms it is important to make a somewhat artificial division of the different forms of diphtheria. It is well to emphasize that diphtheria at the onset is a local disease, and that the constitutional symptoms depend absolutely upon the amount of toxin generated by the specific organism.

### Different Forms

The different forms of diphtheria are: pharyngeal, which is by far the most common and the one usually seen; laryngeal, incorrectly termed "membranous croup;" nasal. cutaneous; diphtheria of the lungs, and finally diphtheria of the eyes.

### The Symptoms.

Having been summoned to treat a child, whom you find sitting around rather listless or in a recumbent posture, you elicit the following history: a feeling of chilliness not amounting to a distinct rigor, headache not particularly severe, and a rise in temperature not particularly marked, say, 100 degrees to 102 degrees, with slight acceleration of the pulse and some feeling of drowsiness and lassitude; soreness in the throat at first slight, but at the end of twelve hours more pronounced. If the fauces are examined at this stage we find a peculiar congestion of the tonsils and adjacent parts, which have a distinctive almost purplish hue. Here and there, especially in the follicles of the tonsils, we often note tiny specks of a grayish-white color. The tongue is usually coated with a dark brownish or grayish fur, through which the papillæ show rather prominently.

### Treatment.

If it is our good fortune to be summoned at this time, what is our duty? Remembering that prevention is far greater than cure, the patient should be promptly put to bed and isolated from the remainder of the family, in the usual manner with which we are all familiar. Having taken a culture of the throat, in which care is exercised to penetrate deeply into the follicles of the tonsils, we should then proceed to treat every case as though we were sure it was diphtheria; otherwise we are not doing our full duty by the patient, for in so doing we can often entirely abort and at other times at least diminish the rapidity of the growth of the organisms in a large percentage of these cases.

Medical treatment consists in giving fractional doses of calomel followed by a purgative, and some pleasant antipyretic and diuretic mixture containing such drugs as potassium citrate, aconite and nitrous ether; and, if bronchitis is present, as is often the case, add some ammonium chloride. Bathe the throat well with chloroform liniment every hour or two.

### The Important Remedy

But the most effective part of the treatment is the local application to the entire throat and especially to the tonsils of the LIQUOR CHLORI COMPOSITUS, of the U. S. P., which contains when freshly prepared about 0.4 per cent. of free chlorine. It should always be freshly prepared, kept tightly corked and in a cool place, and should be dispensed in a dark-colored bottle. A small amount of the solution should be placed in a cup, and then by means of a flexible wire-handled throat brush applied to the throat in its undiluted state. Use thoroughly every hour until relieved and then less frequently. In a large percentage of these cases this treatment will completely arrest the disease, so that, when you call the next day, even if your report from the culture is positive, you will find the child practically well as far as the throat is concerned, and no further treatment will be necessary, not even the use of antitoxin.

### The Use of Antitoxin

However, if the culture is positive, and we find a more decided growth on the throat, as evidenced by patches of membrane of a dirty white color, then antitoxin should be promptly administered and in the proper amount; for I feel convinced that the reason why so many practitioners fail to obtain satisfactory results from antitoxin is due to insufficient dosage. Small children, two to four years of age, give at least five thousand units; older children, eight to ten thousand; and repeat in 12 to 24 hours if necessary. The earlier the antitoxin is administered the better, and the less danger of sequelæ or complications. The duration of the attack of diphtheria has a very important bearing on the quantity to be administered, but always give sufficient to produce the characteristic effect of the serum, viz., shriveling of the membrane, diminution of the nasal discharge, a correction of the fetid odor, and general improvement, no matter if it takes fifty thousand units. If, however, we have not been summoned for 36 hours, or about 24 hours after the appearance of the congestion in the throat, the membrane will have extended very rapidly and covered the tonsils, uvula and the hard and soft palate. Dysphagia now becomes a prominent symptom; the temperature has risen two or three degrees and prostration commences to be marked. The breath of the patient is most distinctive and has a strong septic odor, and often can be recognized several feet away.

### Grave Cases

Now what is our duty, as conditions have become quite grave? The above treatment should be administered promptly and vigorously, including the immediate injection of massive doses of antitoxin, and proper stimulation of the heart, as indicated.

### Uncommon Forms

The symptoms and treatment of the other forms of diptheria, which fortunately are comparatively rare, can be found in all standard works on medicine, and I shall not attempt to discuss them here, as my chief desire has been to emphasize the great value of free chlorine in preference to all the other antiseptics which are applied locally, and which have been recommended for the common or pharyngeal type of diphtheria. Various antiseptics containing solutions of iodine, silver, phenol, iron, boric acid, lime, thymol, peroxide, lactic acid, Dobell's solution and various proprietary antiseptics each have their respective advocates; but I believe the compound solution of chlorine of the

United States Pharmacopœia (freshly prepared) to be the most effective germicide in the entire materia medica for the local treatment of this dangerous and rapidly fatal disease.

---

### THE CASE AGAINST NON-MEDICAL ANESTHETISTS.

LAWRENCE IRWELL, M.A., B.C.L., Buffalo, N. Y.
(The Medical Times, New York City.)

My article entitled, "Non-Medical Anesthetists and the Law," which appeared in a former issue of the "Medical Times" brought me so many letters, chiefly from physicians, but a few from lawyers, that an additional article seems necessary to make certain points clear.

Whether the administration of a general anesthetic by a non-medical person by order of a registered physician and in his presence constitutes the practice of medicine or not depends upon the definition of what constitutes the practice of medicine in the statute of the particular State concerned, unless another statute makes provision for non-medical anesthetists. I have studied the statutes of this State only, but, as mentioned in my last article, the attorney-general of Ohio has given an opinion that the employment of non-medical anesthetists in Ohio is illegal. Since that opinion was written, however, the administration of general anesthetics by dentists for non-dental operations has been legalized in Ohio by statute, a procedure which is against the public interest because to administer a general anesthetic without unnecessary danger to the patient involves a complete medical education including a thorough knowledge of not only physiology, but also pathology. A few dentists may have this knowledge, although the law does not require it, but the Ohio statute now permits any dentist to give any general anesthetic for any operation. The attorney-general of Ohio evidently cares little for enforcing his ruling, otherwise he would have used pressure to induce the State Medical Board to compel the superintendent of the Lakeside Hospital in Cleveland to stop employing nurses to give nitrous-oxide-oxygen, and also to cease "training" nurses to become "specialists" in the administration of this anesthetic which is, for major surgical operations, highly dangerous. The alleged "training" occupies from four to six months! These nurses are taught to give nitrous-oxide-oxygen only, although anybody who knows anything about anesthesia is well aware that as cases occur in which change of anesthetic during an operation is sometimes necessary to save the patient's life, a so-called "trained" anesthetist who can give only one anesthetic is almost as great a menace to the public as a "trained" surgeon whose knowledge of anotomy was limited to the abdomen and its diseases would be. Published statements emanating from Cleveland which declare that many thousand surgical operations have been performed with $N^2O+O$ without a death directly due to the anesthetic should not be taken too seriously because deaths which certain surgeons regard as not directly attributable to the anesthetic might be regarded by some professional anesthetists as a direct result of using $N^2O+O$ as a routine anesthetic. Every professional anesthetist chooses for each operation the particular anesthetic which is most suitable for each case. To argue that a dentist or a nurse can do this is to argue that a dentist or a nurse can, among other matters, ascertain the cardiac condition and the renal condition of the patent.

Except in emergency cases professional medical anesthetists suitably treat their patients prior to operation. If Mr. Ansley Wilcox, honorary counsel of Buffalo General Hospital, is right in his opinion that a nurse can lawfully administer a general anesthetic to a patient in the absence of a registered physician, which I, of course, deny, the day is probably in sight when a surgeon at the hospital named will permit his nitrous-oxide-oxygen "specialist" to anesthetize "private" patients and have them ready to be operated upon when he arrives at the hospital. However, the rules of the hospital referred to prohibit the employment of non-medical anesthetists in the case of "hospital" patients. Notwithstanding opinions to the contrary, and in spite of the absence of any specific contract between a "private" patient and a hospital, the relatives of a "private" patient who died from anesthesia could properly bring suit against any hospital which permitted a nurse to give general anesthetics, unless the patient had been informed prior to the operation that he would be anesthetized by one who was not a registered physician, and his consent obtained. The out-of-date doctrine that however high a price a patient pays for a room in a hospital, that patient cannot sue the trustees on account of a hospital not being a trading institution existing for money-making purposes was disproved by the prolonged proceedings in the case of Helen Ward versus St. Vincent's Hospital, New York. The trustees of a hospital are required to see that unlawful acts do not occur on their premises, and the practice of medicine by medical students and nurses is an unlawful act. In those cases in which the patient or his relatives make arrangements for care at a hospital in a private room with the representative of the hospital, the surgeon not being used as an intermediary in the negotiations, the defence of no contract on the part of the hospital would be mere pettyfogging. Nevertheless, there may be devices by which a hospital might escape paying damages for its negligence if a "private" patient should die as the result of a general anesthetic administered by a nurse, and for that reason, apart entirely from the necessity of protecting the public before unnecessary deaths occur, the law of the State of New York should be made perfectly plain. Writing on August 31, 1914, Mr. Almuth C. Vandiver, counsel of N. Y. County Medical Society, said: "In my opinion this important matter (administration of general anesthetics) should be presented to the legislature at the coming session and speedy action thereon strenuously urged in order that there may be no further doubt as to the identity of the persons who may legally, properly and safely administer to the public."

Mr. James Taylor Lewis, counsel of N. Y. State Medical Society in Journal of that Society, December, 1911:

"I believe that the law does not authorize her (a nurse) to take part in so important, indeed so vital, a procedure incident to surgical work" (as the administration of general anesthetics).

Mr. Frank B. Gilbert, Chief of Law Division of Univ. of State of N. Y. (letter dated February 10, 1914).

"If acting under the advice and direction of a physician and in his presence as an aid to him in the performance of an operation a trained nurse administers an anesthetic, she will be deemed to have done so under the personal supervision of the physician and the act will be his."

The opinion given by Mr. Wilson M. Powell, counsel of New York Hospital, New York, coincides with that of Mr. Lewis, but that of Mr. Wesley C. Dudley, district attorney of Erie County, is very different and agrees with Mr. Gilbert's opinion. On May 13, 1912, Mr. Dudley wrote: "It would seem to me that a nurse who administers an anesthetic in the presence of and under the direction of a duly licensed and practicing physician or surgeon, would hardly come within the definition of practicing medicine."

My impression is that neither Mr. Gilbert nor Mr. Dudley can ever have witnessed the administration of a general anesthetic for a surgical operation, although they may both have been operated upon. The average surgeon does not—and cannot—"coach" his anesthetist; he takes no more notice of that anesthetist than if he (or she) were not in the room. The surgeon's "main object for the moment is to secure cadaveric relaxation of the subject" (Dr. Wm. C. Woolsey). A surgeon who can instruct an anesthetist and operate as he should operate is a genius. That he exists is possible, but very improbable.

My own way of settling the legal status of non-medical anesthetists would be to prosecute one of them in the city of Buffalo, but this procedure is impossible at present as the district attorney of Erie County, as mentioned above, holds that they do not practice medicine. Both this learned gentleman's opinion and that of Mr. Gilbert involve one of two hypotheses, viz: either that the surgeon instructs the anesthetist as to the amount of anesthetic which must be given at any particular moment, and later increased or decreased, or that a surgeon can lawfully delegate to a non-medical person his legal right to practice medicine, because prescribing constitutes practicing medicine according to the law of the State of New York. Both hypotheses are fallacies. During the administration of a general anesthetic conditions sometimes arise which necessitate the use of a drug. When a "specially trained" nurse-anesthetist chooses the drug and decides upon the dose, she practices medicine in violation of the law of the State of New York. If the opinions of Mr. Gilbert and Mr. Dudley are good law, and a registered nurse can lawfully administer a general anesthetic in the presence of and by order of a registered physician, she can equally lawfully perform a surgical operation in similar circumstances. She is forbidden by statute to diagnose; treat and prescribe every bit as much as she is to operate. If a "specially trained" nurse, registered in New York State, can lawfully administer a general anesthetic, then any registered nurse can equally lawfully administer any general anesthetic, although she may not have had any instruction in the art of giving anesthetics, and although she may not know what $N_2O+O$ means, and although she may never even have seen the complicated apparatus by which it is given. If the legislature in creating the status of registered nurse had intended to confer upon trained nurses the legal right to administer general anesthetics, it would have required them to receive instruction in the art of administering anesthetics. This is not the case. They are, however, specifically forbidden to practice medicine, or "to undertake the treatment or cure of disease." What possible excuse can be given for relegating any act so dangerous as the administration of a general anesthetic to a nurse when no reliable physician would permit a nurse to write a prescription for diarrhoea, or even for a "cold?" The usual excuses are that the expense to the patient is less, that the nurse's services can always be secured at short notice, and that the attention is given to the anesthesia exclusively, while some physicians watch the operation although giving the anesthetic. The answers are that to "private" patients the difference in cost is too small to be important. Moreover, many druggists will often unlawfully prescribe for disorders, charging merely for the medicine, at a price considerably below a physician's fee for an office call. Again, the administration of anesthetics is a specialty in medicine which in large cities is in the hands of trained specialists who are not interested in surgical work. Even general practioners who give many anesthetics in the smaller cities and in the country do not watch the operation if they know anything worthy of the name of the art of anesthesia. I suspect very strongly, however, that before I drew attention to this important subject some surgeons were paying nurses salaries and were changing their patients sufficient for each anesthesia to make a handsome profit out of this procedure. I admit, nevertheless, that I cannot prove this suspicion as the evidence is insufficient.

Why do not those nurses who wish to make a specialty of giving anesthetics go to a medical school and obtain a medical education? The usual reason is that although they pose as experts in a medical specialty of a highly technical character, they are not sufficiently equipped mentally to absorb elementary medical knowledge; and the same reason explains why a few dentists in New York who try to practice as medical specialists in the administration of general anesthetics, but who are not physicians, do not secure the legal right to put M. D. after their names. Unless the legislature interferes very soon, the day is not far distant when some nurses will advertise in small places that they are specialists in giving anesthetics, and that they are willing to administer anesthetics for any physician. Already some of the ladies are claiming that to administer an anesthetic is a part of the practice of nursing! Is it not about time that in the public interest the National Society of Anesthetists as well as the State Society should make a determined effort to obtain such legislation as will protect men and women who know nothing of the dangers of anesthesia from the dire effects of being anesthetised by an amateur, whether dentist or nurse? As long ago as May 8, 1914, Mr. Almuth C. Vandiver, in a paper read to the New York State Society, urged that the legal status of nurses administering anesthetics should be fixed by the legislature in the instance of that Society. No assistance can be expected from the Regents or from any department of the State at Albany. Writing on March 4, 1914, Mr. Frank B. Gilbert, Chief of the Law Department of the Regents, said: "The Regents of the University have assumed the attitude of not proposing legislation by way of amendments to the medical practice act without such legislation being proposed and supported by the medical profession as a body." I cannot agree with this statement. The bills introduced in 1913 and 1914 by the State Education Department, which proposed to take away the license to practice medicine of any physician who was guilty of unprofessional conduct, although exactly what constitutes unprofessional conduct was not made perfectly clear, were by no means "supported by the medical profession in a body." Both bills were very properly defeated: that of 1913 was undoubtedly unconstitutional. Mr. Gilbert continues, "We are able usually to ascertain the sentiments of the profession through recognized organizations. I am quite sure that the Regents have had no request from any organizesd body of physicians that a bill be prepared and introduced at the initiative of the Regents relative to the administration of anesthetics as a part of the practice of medicine. * * * The only point is whether or not the Regents are required or warranted in proposing legislation along the line indicated by you." The same attitude was assumed in March, 1914, by Dr. Downing, Assistant Commissioner of Education. Now, what does this attitude mean? It means simply that in the opinion of Mr. Gilbert and Dr. Downing medical laws in the State of New York exist, not for the protection of the public, but solely for the protection of the medical profession. If, then, physicians as a body wish to employ as anesthetists persons who are not required by law to receive education in the administration of general anesthetics, so much the worse for suffering humanity; the Regents must not interfere, because medical men as a whole do not favor legislation on the subject! Is this doctrine consistent with government of the State for the benefit of the people? Certainly not, but, as a fact, the consensus of opinion among reputable and conscientious physicians is against non-medical anesthetists.

What the attitude of the New York State Medical Society is toward legislation making clear the fact that the administration of a general anesthetic in the presence of and by order of a physician constitutes the practice of medicine, and not the practice of nursing, I do not know specifically. Writing to me on April 17, 1914, Dr. Lewis K. Neff, of New York City, said: "I might venture the opinion that the Medical Society of the State of New York would scarcely look with favor upon any further amendments to Article VIII of the Public Health Law. There have been many various attempts to amend this article, and thus far I hope we have succeeded in preventing their success. However, should you consider it desirable, if you will send me your copy of the proposed legislation, I should be very glad to read it over." Dr. Neff is chairman of committee on legislation of New York State Medical Society. A copy of the proposed legislation was mailed to him on April 20, 1914. It makes no attempt to amend any statute or to add anything new to any statute. The sole object of the proposed statute is to clarify the existing law, and to make plain the obvious intention of the legislature. Since the date last named, I have not heard from Dr. Neff. I now draw attention to two excerpts from the Dietetic and Hygienic Gazette of New York, which, when taken together, are very illuminating:

D. & H. Gazette, January, 1912, p. 50. Dr. Babcock, president of American Hospital Association:

"The lack of proper material for pupils in our training schools (for nurses) is so great that it has become necessary in most schools to accept for training material lacking a good English education and much of the fundamental training of life."

D. & H. Gazette, December, 1913, p. 573. Dr. Tinker, of Ithaca, N. Y.:

"All those here present who have been obliged to take a general anesthetic appreciate the importance of care in its administration. Skill in anesthesia is gained only by much experience and the anesthetist should be in constant practice. The importance of the anesthetist's position has been estimated by several of our best known surgeons as second only to that of the operating surgeon himself. . . . I believe that there will be in the future many thousands of trained nurses in the smaller cities who will devote themselves to the administration of anesthetics alone."

Dr. Tinker is, I believe, a surgeon who makes a specialty of operations for goitre. If this is correct, he must have had some experience of trained physicians who make a specialty of anesthesia, and consequently he must know the immense difference between the trained anesthetist who is a physician and the anesthetist who is not. He referred to over 1,000 operations in the past four years "in our little operating room with a death-rate less than one in five hundred major operations during the past two years." How many of these deaths were due to the anesthetic he did not say, nor did he mention that if the surgeon is competent and his technique up to date, when death occurs as the result of the operation, the anesthetic or the anesthetist has usually been to some extent to blame. Dr. Tinker admits the paramount importance of the anesthetist's position, but believes that nurses can fill it satisfactorily, although he must realize that the mental equipment necessary for a thoroughly safe anesthetist is far above that possessed by the average nurse. Is that surprising when "most schools" three years ago were accepting for training "material lacking a good English education and much of the fundamental training of life?" In ordinary, uncomplicated cases the administration of a general anesthetic may appear to be a comparatively simple matter. After it, the patient may suffer from much nausea and some vomiting, but recovers, and no more is heard about the case. The whole course of events, however, may point to ignorance or inexperience on the part of the anesthetist which, in an exceptional case, would be liable to cause a fatal termination. Without fear of contradiction by any competent person, I assert that a large proportion of non-medical anesthetists believe that the only serious danger of death from anesthesia is due to insufficient oxygen, and that, speaking generally, they do not know what acapnia means. Yet death from acapnia is far from impossible.

Writing to me on January 16, 1914, Dr. John B. Murphy of Chicago, said: "I do not know what the law is in Illinois. I do know, however, that for the last sixteen or seventeen years my anesthesias have been given by a Sister. I believe she is the best anesthetist I have even seen. She is not a medical graduate and knows nothing about medicine, except she was a trained nurse. She knows all about the practical administration of anesthetics, however. In the entire period of time that she has been with me, we have not had an untoward condition, and I dare say she would now approximate about 20,000 anesthesias." Dr. Murphy may perhaps never have seen a medical specialist on anesthesia give an anesthetic, and he may be comparing his anesthetist with interns. He would not ask me to believe that the most experienced midwife in Chicago or New York is as expert as a physician who makes a specialty of obstetrics. Imagine any one comparing a midwife who has attended many confinements, with Cragin of New York or Blacker of London! Yet assuming that Dr. Murphy has seen Gatch, Gwathmey, Bennet or Woolsey give an anesthetic, he professes to believe that his non-medical anesthetist is their equal in skill in the administration of general anesthetics! But he says that she "knows nothing about medicine, except that she is a trained nurse." If that is so, she may be ignorant of the meaning of "scyanosis" and of "acapnia" as these terms are not connected with the practice of nursing. Dr. Murphy has not had "an untoward condition" in "the last sixteen or seventeen years," but he does not explain what those words mean.

Dr. Murphy says that he does not know what the law is in Illinois. If the practice of medicine in that state is still controlled by the statute which was in force in 1909, or if the definition of what constitutes the practice of medicine is identical with section seven of the statute then in force, in my opinion, the administration of a general anesthetic for any non-dental operation by any person other than a licensed physician, constitutes practicing medicine and is a violation of the law of Illinois.

Dr. George H. Simmons, editor of the Journal A. M. A., writing to me on October 18, 1912, informed me that although "a trained anesthetist is required, a broad knowledge of medicine is not regarded as necessary." He omits to relate, however, by whom it is not so regarded. I vetnure to assert that more than ninety per cent. of the well educated physicians in the United States are of the opinion that a broad medical education is necessary for anesthetists for the very good reason that the patient's life is in danger every moment that he is under the influence of a general anesthetic. When a man or woman is as near to death as he or she is when fully anesthetised, do not ordinary caution and ordinary common sense demand that a thoroughly trained physician ought to be in control of the patient and the anesthetic—and not a dentist, a nurse, or an inexperienced intern? Dr. Simmons tells me that "in any event," the operator is the one who as responsible if death occurs."

Dr. Simomns says: "The British Medical Association had a commission which worked for many years and made voluminous reports, but so far as I know there was nothing in any of the reports to warrant forbidding the giving of anesthetics by non-medical women." Being skeptical, I communicated with Dr. Dawson Williams, editor of the British Medical Journal. He kindly procured for me a statement from a great professional anesthetist, Sir Frederick Hewitt of St. George's Hospital, London. It is refreshing to read what the latter says:

"There is a general feeling amongst the surgeons of this country against entrusting the administration of general anesthetics to nurses, except, perhaps, in obstetric cases in which the medical attendant is single-handed and is therefore obliged whilst applying forceps, for example, to allow the nurse to continue the anesthetic under his supervision. So far as I am aware the practice of allowing nurses to administer anesthetics in general surgery is non-existent in England. There may possibly be a few instances in which dentists allow nurses, or other female assistants, to administer nitrous oxide for them; but the fact that the General Medical Council, the British Medical Association, the Royal College of Surgeons, the British Dental Association, the British Association for the Advancement of Science, and the Medico-Legal Society, have one and all endorsed the recent proposal to place the administration of every general anesthetic in the hands of a qualified medical or dental practitioner is sufficiently indicative of the views generally held by the profession in regard to anesthetics. There are now, indeed, few if any, hospitals at which the anesthetics for major operations are given by any but specially trained medical men or women. We have quite given up the practice of regarding the operator as responsible for the anesthetic, now that the latter is almost invariably administered by a qualified and properly trained person. At St. George's Hospital the requirements of the surgeons are such that there is an increasing demand for highly skilled anesthetists, and as it is my function as head of the Anesthetic Department at that Hospital to endeavor to meet such requirements by proper organization, I am i na position to state very definitely that any omvement towards a lower level of efficiency would be regarded as dangerous and retrogressive."

The late Dr. A. J. Bristow, of Brooklyn, was much opposed to non-medical anesthetists, and no one will deny that he was a surgeon of considerable experience. In a letter dated Jan. 17, 1913, he said: "This rank impropriety (the employment of nurses as anesthetists) will go on until somebody dies on the table and the patient's friends find it out and bring suit for damages. The whole affair is improper and cannot be defended by any such analogy as Dr. B. suggests. It is one thing when you are in the back woods, and can get no other assistance, to ask a layman to hold an ether cone—quite a different proposition to encourage the improper use of unauthorized persons in a public hospital."

At the next (1916) session of the legislature of this state (New York) an attempt will be made to obtain legislation which will provide that the administration of general anesthetics is a part of the practice of medicine. The proposed addition to the law, of which a copy is appended, will not interfere with the lawful rights of dentists, with the employment of any reliable person in an emergency, or with the instruction of medical or dental students.

A Bill to Regulate the Administration of General Anesthetics by Persons other than Licensed and Registered Physicians.

The administration by any process of any substance, liquid or gas, commonly called a general anesthetic, for the purpose of producing unconsciousness, shall constitute the practice of medicine and surgery, even if undertaken in the presence of and by order of a licensed and registered physician, surgeon or dentist, with the following exceptions:

1. A licensed and registered dentist may administer any general anesthetic for any operation which a dentist can lawfully perform, but not for any operation which a dentist cannot lawfully perform, even if a licensed physician is present and directs such dentist to administer such general anesthetic, except as is hereinafter provided.

2. In any case of sudden or severe illness or sickness in which the licensed physician or surgeon in attendance upon the patient conscientiously believes that the delay necessary to obtain the services of another licensed physician to administer a general anesthetic will endanger the life of the patient, that attending physician or surgeon may employ any reliable person to administer such general anesthetic in his presence and directly under his instructions; but not in his absence. The employment in this manner of any person other than a licensed and registered physician to administer any general anesthetic must be strictly limited to emergencies in which the services as anesthetist of a licensed and registered physician acnnot be quickly and easily obtained, and must not be a regular or habitual practice.

Nothing contained in this Statute shall be construed to prohibit necessary practical instruction in the administration of general anesthetics being given to registered medical students by licensed physicians, or to registered dental students by licensed dentists or licensed physicians; always provided that while giving such instruction the instructor devotes his entire attention to teaching and neither operates nor does any medical, surgical or dental work unconnected with the administration of a general anesthetic.

Since the above was written, I have seen an article by Dr. Woods Hutchinson in "Good Housekeeping" for last June, entitled "The Community Hospital." Dr. Hutchinson says, on p. 90: "One of the greatest obstacles, however, to the proper administration of anesthetics in adequate amounts in labor * * * is that it is a delicate and somewhat risky performance, requiring either the services of a nurse who has had special training and skill in anesthetization or else the almost continuous presence of the doctor himself."

Dr. Hutchinson evidently considers that nurses should be permitted to anesthetize obstetrical patients in the absence of a physician, although he calls the process "delicate and somewhat risky." I am assured that the delivery of the infant in normal cases is neither delicate nor risky, so that, according to Dr. Hutchinson's ideas, the dangerous part of an ordinary obstetrical case may be left to a non-medical individual, and the simple part to a physician. That any one will treat this proposition seriously seems incredible, although a properly educated trained nurse might be a much safer obstetrician than are many midwives.

425 Porter Avenue.

### ABSTRACT OF CLINICAL LECTURE IN THE LONG ISLAND HOSPITAL ON THE DIAGNOSIS OF SYPHILIS BY LABORATORY METHODS.

By Henry H. Morton, M. D.

Clinical Professor of Genito-Urinary diseases in the Long Island Hospital; Genito-Urinary Surgeon to Long Island College and Kings County Hospitals and the Polhemus Memorial Clinic, Etc., Brooklyn, N. Y.

We have four ways of making an examination for the purpose of making diagnosis of syphilis:

1. The demonstration of the spiorcheta in the suspected lesion.
2. The Wasserman reaction in the blood.
3. The cutaneous or Luetin reaction of Noguchi.
4. The findings in the spinal fluid.

Clinical experience must supplement laboratory diagnosis, for sometimes laboratory reports are misleading.

For the demonstraion of the spirocheta the "Dark Field" is the best method as the living spirocheta may be seen.

The India ink and Giemsa stains are also extensively used for the demonstration of spirocheta in smears, while the Levaditi stain is used to demonstrate the spirocheta in tissues.

The Wasserman reaction is depended upon for the diagnosis of Syphilis today. The old method is the best, as short cuts are unreliable.

A positive reaction appears 6-8 weeks after infection. 100 per cent. of secondary syphitics untreated give a positive reaction.

In some cases, in spite of good treatment, it is impossible to change th ereaction, if, however, it is reduced but is not negative it shows the need of more treatment.

In suspected cases where the Wasserman is negative a small dose of Salvarsan or Mercury will often cause the reaction to be positive in 24-48 hours. This is called a provocative Wasserman.

The Luetin reaction was devised by Noguchi in 1911. A mixed culture of killed spirochetae are injected beneath the skin. A positive reaction appears as a papule or pustule appears in 3-5 days. Nichols and Craig found that the positive reaction appeared more consistently in latent cases.

The findings in the spinal fluid are useful in diagnosing cerebro-spinal syphilis. There is very little danger in lumbar puncture if properly done.

In examining the spinal fluid we estimate the pressure, make a cellocunt. estimate the globulin and do a Wasserman.

Up to 5 cells per cm is normal, 5-10 is doubtful, and over 10 is pathological. Increased cell count shows irritation along the cord.

Globulin is not normally found in spinal fluid, and its presence shows irritation. It is present in 80-100 per cent. of syphitic nervous disease.

In syphilitic nervous disease the Wasserman reaction is usually present. In Tabes about 60-70 per cent. positive. In Paresis 100 per cent. positive.

All laboratory reports must come from reliable laboratories, else reports will be misleading and then clinical experience must determine the diagnosis.

---

### THE NITROID CRISIS OF MILIAN; THEIR SYMPTOMS AND TREATMENT.

By B. Barker Beeson, M. D., Assistant in Cutaneous Pathology, Rush Medical College, Chicago, Ill.

(The Urologic and Cutaneous Review, September, 1915.)

The intravenous administration of Salvarsan and Neosalvarsan is not usually followed by untoward symptoms, but occasionally, more often after "606," the patient exhibits phenomena which are both alarming and dangerous. It was my good fortune to observe several cases of this sort while working in the service of Milian, at La Rouchefoucauld, Paris. Believing that a better knowledge of them would be of value, I shall briefly outline the symptoms which may be encountered and tell how they should be treated.

These attacks have been most thoroughly studied by Milian who, from their resemblance to the congestive phenomena produced by the inhalation of amyl nitrite, gave them the name "crisin nitritoide." The onset of such a crisis is very sudden often during an intravenous injection or soon thereafter. The patient's face becomes markedly flushed, the conjunctivae are injected, the lips and tongue may swell, marked chilliness with rigors is noticed, perspiration is profuse, and usually nausea followed by vomiting helps to complete the picture. Both in character and duration the attacks differ greatly, some being accompanied by severe headache, epiphora, difficult breathing with intense, even agonizing, pain in the precordium or epigastric region. Fever and diarrhoea are not uncommon. The pulse is usually rapid. When severe such an attack may end in syncope. Somewhat similar attacks occurring later, on the third or fourth day are known as secondary nitroid crisis. They are milder than the usual type.

A condition known as serous apoplexy has also been noted after intravenous administration of salvarsan and neosalvarsan. It is said by Milian to be analogous to the nitroid crisis, save that it affects the brain. In this all goes well for three or four days after the injection. Suddenly the patient becomes very agitated, is taken with epileptiform seizures, both clonic and tonic, which become more and more frequent. Later coma ensues followed by death on the fourth day, in most cases. At autopsy these cases have shown marked engorgement of the lateral ventricles and cerebrospinal canal, while fatal cases of nitroid crisis have presented intense congestion of all the viscera.

Many of those intolerant to "606" and "914" notice a premonitory aura, according to some clinicians, which may occur during the course of the injection or soon after it. This consists of a peculiar taste said to resemble that of ether, salt or spices. It is said by those who have observed this aura that it does not occur in patients who tolerate the products of Ehrlich.

Insufficient action of the adrenal glands plays the leading role in these attacks since in almost all cases they respond to adrenalin or suprarenin, a synthetic product. The vaso-constrictive action of these agents seems to bridge over the gap most effectually. A purely constrictive action upon the blood vessels is not, however, all that is required since ergotin, a marked vaso-constrictor, does not act nearly so well as adrenalin or suprarenin. Hypophysin has also been given but with relatively mediocre results. Perhaps adrenalin and suprarenin act in a cardio-tonic manner as has been suggested by several writers.

Adrenalin can be given subcutaneously, intramuscularly or intravenously. The first named method permits of a too rapid absorption, the last one is only reserved for grave cases, especially serous apoplexy, while the intramuscular route is the one most generally employed. It permits of a more durable action. The minimum dose is one milligram, equal approximately to one cubic centimeter of a 1:1000 solution. In cases of marked crisis even four milligrams can be given. The adrenalin solution should be given about 10 minutes before the injection is begun. The muscles of the thighs or buttocks are the best sites.

When a prolonged action is desired one can, in addition to the adrenalin which has been injected, give it in pill form. Each pill contains one-fourth milligram and six can be given daily, two in the morning, the same number at noon and two at night.

Should a crisis occur during an injection one should at once stop and give adrenalin as above described. A careful attention to dosage employed as well as to the interval between injections which should be at least a week, is also essential. When using salvarsan one should be certain to add a sufficient amount of soda to the solution since it has been shown that a slightly acid mixture of salvarsan can easily cause a crisis.

The action of adrenalin in these cases is undoubted. I have on several occasions seen such an attack begin and then after adrenalin had been given, cease almost as if by magic. Those who have patients intolerant to "606" and "914" will do well to give this treatment a trial.

Just a word about the after results of adrenalin treatment, especially when injected. It is usually followed within a few minutes by marked pallor, fast pulse, 100-120, pain at the site of injection, and occasionally generalized trembling. These, however, soon pass over.

My sincere thanks are due Dr. Gaston Milian for his courtesy in permitting me to follow the treatment of a number of these cases.

802 West Madison Street.

---

### THE TREATMENT OF ARTHRITIS.*

By Dr. E. Seaborn, London, Ont.

To understand joint diseases we must understand the capacity for repair of the different constituents of a joint, that is, the bone and capsule (continuous with each other) and the cartilage and synovial membrane (continuous with each other).

First, the bone has a very great capacity for withstanding attack, being able to enlarge, if necessary, to surround and encapsulate a focus or irritation; is able to form new bone and so compensate any loss of substance.

The capsule is one of the most resistant of the tissue and persists through any irirtation, even though it has no power of regeneration. The cartilage is incapable of any enlargement or of any regeneration, having no direct blood supply. It is dependent on the subjacent bone, and is destroyed by irritation of that structure early in any disease.

The synovial membrane is capable of very great hypertrophy, but the new tissue is very largely formed of small white cells resembling granulation tissue and having the same phagocytic action.

